# Primary Nasal Epithelial Cells as a Surrogate Cell Culture Model for Type-II Alveolar Cells to Study ABCA-3 Deficiency

**DOI:** 10.3389/fmed.2022.827416

**Published:** 2022-02-21

**Authors:** Nicole C. Shaw, Anthony Kicic, Sue Fletcher, Stephen D. Wilton, Stephen M. Stick, André Schultz

**Affiliations:** ^1^Faculty of Health and Medical Sciences, The University of Western Australia, Perth, WA, Australia; ^2^Wal-yan Respiratory Research Centre, Telethon Kids Institute, The University of Western Australia, Perth, WA, Australia; ^3^Centre for Cell Therapy and Regenerative Medicine, School of Medicine and Pharmacology, The University of Western Australia, Perth, WA, Australia; ^4^Department of Respiratory and Sleep Medicine, Perth Children's Hospital, Perth, WA, Australia; ^5^Occupation and Environment, School of Public Health, Curtin University, Perth, WA, Australia; ^6^Centre for Neuromuscular and Neurological Disorders, Perron Institute for Neurological and Translational Sciences, The University of Western Australia, Perth, WA, Australia; ^7^Centre for Molecular Medicine and Innovative Therapeutics, Murdoch University, Perth, WA, Australia

**Keywords:** ABCA-3, nasal cells, alveolar cells, surfactant, diffuse lung disease

## Abstract

ATP Binding Cassette Subfamily A Member 3 (ABCA-3) is a lipid transporter protein highly expressed in type-II alveolar (AT-II) cells. Mutations in *ABCA3* can result in severe respiratory disease in infants and children. To study ABCA-3 deficiency *in vitro*, primary AT-II cells would be the cell culture of choice although sample accessibility is limited. Our aim was to investigate the suitability of primary nasal epithelial cells, as a surrogate culture model for AT-II cells, to study ABCA-3 deficiency. Expression of *ABCA3*, and surfactant protein genes, *SFTPB* and *SFTPC*, was detected in primary nasal epithelial cells but at a significantly lower level than in AT-II cells. ABCA-3, SP-B, and SP-C were detected by immunofluorescence microscopy in primary nasal epithelial cells. However, SP-B and SP-C were undetectable in primary nasal epithelial cells using western blotting. Structurally imperfect lamellar bodies were observed in primary nasal epithelial cells using transmission electron microscopy. Functional assessment of the ABCA-3 protein demonstrated that higher concentrations of doxorubicin reduced cell viability in ABCA-3 deficient nasal epithelial cells compared to controls in an assay-dependent manner. Our results indicate that there may be a role for primary nasal epithelial cell cultures to model ABCA-3 deficiency *in vitro*, although additional cell culture models that more effectively recapitulate the AT-II phenotype may be required.

## Introduction

Adenosine triphosphate binding cassette sub-family A member 3 (ABCA-3) is a membrane-bound, transporter protein highly expressed in AT-II cells in the lung. The ABCA-3 protein is present at the limiting membrane of lamellar bodies in AT-II cells and is responsible for the transport of surfactant lipids from the cytoplasm into the lamellar body (1). Exocytosis of the contents of lamellar bodies at the alveolar surface forms the surfactant layer at the air-liquid interface (2), a layer with biophysical properties that reduce surface tension in the lungs, thereby preventing atelectasis.

Homozygous or compound heterozygous mutations in the *ABCA3* gene can lead to a loss of ABCA-3 function resulting in severe respiratory disease that can be fatal (3). The disease, known as ABCA-3 deficiency, is rare and can present either during the neonatal period with respiratory distress or later during infancy and childhood with symptoms associated with diffuse lung disease (4). Over 400 mutations in *ABCA3* have been reported to date (5) with the majority of mutations classified as private (6). Some of the disease heterogeneity seen in ABCA-3 deficiency can be accounted for by the diversity of the individual mutations (7).

Currently, there are few therapeutic options for patients with ABCA-3 deficiency. Hydroxychloroquine and corticosteroids are widely used to treat lung disease but do not always improve clinical outcomes (8) while the risks of their long-term use are well-documented (9, 10). Lung transplantation is considered in the most severe of cases but there are issues surrounding the shortage of donor organs as well as the risk of graft rejection and post-operative complications, not to mention the life-long immunosuppressive therapy that will be required (11). Therefore, there is a need to develop new, effective therapeutics for patients with ABCA-3 deficiency.

To test candidate therapies, a biologically-relevant *in vitro* cell culture model is required. Cell cultures derived from patients with ABCA-3 deficiency that reflect the genetic and phenotypic features of ABCA3-associated lung disease would provide a means to study disease mechanisms and investigate potential therapeutics. Primary AT-II cell cultures, while physiologically relevant to ABCA-3 deficiency, are not a readily accessible cell type to establish *in vitro* cell cultures due to the need for a lung biopsy. The majority of cell culture work to date to investigate ABCA-3 disorders has been performed in cell lines (A549s or HEK293s) genetically modified to stably express *ABCA3* variants (7, 12–14). A limitation to this approach is that the genetic background of the host cell is not identical to the patient and so patient-specific genetic influences may be lost.

The expression of *ABCA3* is not limited to AT-II cells and is in fact expressed across a wide range of tissue types including the nasopharynx (15). There is also evidence that other AT-II cell markers are present in the nasal epithelium (16). Nasal epithelial cells are readily accessible by minimally invasive methods to establish primary airway epithelial cell cultures. Additionally, conditionally reprogramming methodologies for primary epithelial cell culture can overcome some of the limitations of traditional primary epithelial cell culture, increasing the expansion potential of primary epithelial cells (17–19). Furthermore, primary cell culture models also have the benefit over transfected cell lines of being genetically identical to the patient from which the cells are derived.

We hypothesized that primary nasal epithelial cells would be an appropriate surrogate AT-II cell culture model to study ABCA-3 deficiency and aimed to determine whether: (1) ABCA-3 was expressed at a transcriptional and protein level in primary nasal epithelial cells; (2) there was evidence of surfactant biology in primary nasal epithelial cells such as the presence of surfactant proteins and lamellar bodies; and (3) a difference in ABCA-3 function in primary nasal epithelial cells derived from participants, with and without ABCA-3 deficiency, could be measured using a doxorubicin detoxification assay.

## Materials and Methods

### Cell Culture

Primary nasal epithelial cells derived from children with and without ABCA-3 deficiency were collected by cytological brushing of the inferior nasal turbinate as previously described (20). Conditionally reprogrammed epithelial cell cultures were established using isolated nasal cells and an irradiated NIH-3T3 cell feeder layer in F-medium containing 10 μM ROCK inhibitor (17, 18). After expansion of conditionally-reprogrammed epithelial cells, primary nasal epithelial cells were subsequently seeded in feeder-free conditions in BEGM^TM^ Bronchial Epithelial Cell Growth Medium BulletKit^TM^ (Lonza, Basel, Switzerland) to establish cell cultures for downstream analysis and functional studies. Of the two children with ABCA-3 deficiency, Participant 1 was female, aged 2.2 years at the time of sampling and carried a homozygous mutation in *ABCA3* (c.920C>T/c.920C>T). Participant 2 was male, aged 7.5 years at the time of sampling and carried a compound heterozygous mutation in *ABCA3* (c.838C>T/c.3997_3998delAG). Human primary AT-I and AT-II cells were isolated from lung tissue resections, purified and cultured as previously described (21). The Princess Margaret Hospital Ethics committee approved research involving participants with ABCA-3 deficiency while the St John of God Ethics Committee approved research involving participants without ABCA-3 deficiency. All research was performed in accordance with relevant guidelines and regulations. Informed consent was obtained from participants or their legal guardians prior to sampling.

### Digital Droplet Polymerase Chain Reaction (ddPCR)

Primary cell cultures were lysed with RLT lysis buffer (supplemented with 1% (v/v) 2-Mercaptoethanol) and RNA extracted using the Purelink™ RNA Mini Kit (Ambion, Carlsbad, CA, USA) according to the manufacturer's instructions. Quantity and purity of extracted RNA was determined using an ND-100 spectrophotometer and cDNA synthesized using the MultiScribe™ Reverse Transcription Kit (Invitrogen™, Carlsbad, CA, USA) as per the manufacturer's instructions. Sample cDNA was used to prepare 25 μL ddPCR reactions with 12.5 μL ddPCR^TM^ Supermix for Probes (Bio-Rad, Hercules, CA, USA), 1.25 μL TaqMan probe (Applied Biosystems, Foster City, California, United States) specific to ABCA3, SFTPB, and SFTPC and nuclease free water. Droplets were then generated from the reaction mix using a QX200™ Droplet Generator (Bio-Rad, Hercules, CA, USA) and the reactions run in a Bio-Rad T100™Thermal Cycler (Gladesville, NSW, Australia) according to the ddPCR program described by the manufacturer. The concentration (copies/μL) of *ABCA3, SFTPB*, and *SFTPC* in the reaction mix was determined based on the number of positive droplets detected using a Droplet Reader (Bio-Rad, Hercules, CA, USA). Copies per ng of RNA equivalent was then calculated based on the original template load of cDNA.

### Western Blot

Primary cell cultures were lysed with cell extraction buffer and total protein concentration determined using the micro-Bicinchoninic acid protein assay reagent kit (Thermo Fisher Scientific, Scoresby, VIC, Australia) according to the manufacturer's instructions. A 10 μg sample of protein was either treated with 4X Bolt™ LDS Sample Buffer (Thermo Fisher Scientific, Scoresby, VIC, Australia) for SP-B analysis or 4x Laemmli buffer (8% SDS, 20% 2-Mercaptoethanol, 40% glycerol, 0.25 M Tris HCl) for SP-C analysis, for 10 min at 70°C before being loaded into a pre-cast 4–12% Bis-Tris Plus Gel (Thermo Fisher Scientific, Scoresby, VIC, Australia). Samples were then electrophoresed using a Novek Bolt™ apparatus (Life technologies, Carlsbad, CA, USA) in MES SDS running buffer (Thermo Fisher Scientific, Scoresby, VIC, Australia) at a constant 200 V for 15–35 min at room temperature. Proteins were transferred onto a PVDF membrane (Merck, Kenilworth, NJ, USA) using a wet transfer method at 100 V for 2 h at 4°C in transfer buffer (0.3% Tris-base, 1.5% glycine, 20% methanol) before blocking for 1 h at room temperature with Odyssey® Blocking Buffer (LI-COR, Lincoln, NE, USA). Dilutions of rabbit anti-SP-B (1:2,500; Seven Hills Bioreagents, Cincinnati, OH, USA) or rabbit anti-SP-C (1:2,500; Seven Hills Bioreagents, Cincinnati, OH, USA) were prepared in Odyssey® Blocking Buffer and incubated with the membranes overnight at 4°C. Membranes were washed in TBS-T before incubation with goat anti-rabbit IRDye® secondary antibody (1:20,000, Li-Cor, Lincoln, NE, USA) for 2 h at room temperature. Membranes were washed again in TBS-T and incubated overnight with the antibody staining process repeated for the house-keeper protein, β-actin (1:2,500, Sigma-aldrich, Sigma Aldrich, St. Louis, MO, USA). Subsequently, membranes were scanned using the LI-COR Odyssey infrared scanner (Li-Cor, Lincoln, NE, USA) and the integrated density of the protein bands quantified using ImageJ software. The integrated density of each band was normalized to that of the house-keeping protein.

### Immunofluorescence Microscopy

Primary nasal epithelial cells cultured in chamberslides (ibidi, Planegg, Germany) were fixed with 3.7% formaldehyde for 20 min at room temperature before submersion in PBS. The cells were then permeabilized with 0.3% (v/v) Triton-X-100 for 25 min and then blocked with Odyssey® Blocking Buffer (Li-Cor, Lincoln, NE, USA) for 30 min at room temperature. Primary antibodies for ABCA-3 (1:100; Seven Hills Bioreagents, Cincinnati, OH, USA), SP-B (1:500; Seven Hills Bioreagents, Cincinnati, OH, USA), and SP-C (1:100; Seven Hills Bioreagents, Cincinnati, OH, USA) were prepared in Odyssey® Blocking Buffer and incubated overnight at 4°C. Cells were then washed and subsequently incubated with anti-rabbit Alexa Fluor® 488 or anti-rabbit Alexa Fluor® 555 (1:1,000; Invitrogen, Carlsbad, CA, USA) secondary antibodies for 1 h at room temperature protected from light. Cells were washed again and counterstained with DAPI (1:10,000), mounted and visualized using a fluorescence microscope (Nikon, Tokyo, Japan). Matched negative controls without primary antibody were included to determine background fluorescence.

### Electron Microscopy

Primary nasal epithelial cells were detached from the cell culture vessel, harvested by centrifugation, fixed in 2.5% glutaraldehyde and embedded in 10% (w/v) gelatin. The samples were then treated with 1% (v/v) osmium and processed under vacuum in a PELCO Biowave (Ted Pella, Inc., Redding, CA, USA) fitted with a PELCO coldspot for a total of 4 min at 80 W. Samples were dehydrated with increasing concentrations of ethanol followed by 100% acetone in the PELCO Biowave at 250 W for 40 s for each step. Infiltration of samples with Procure-araldite resin progressed through increasing concentrations of resin in acetone for 3 h at room temperature for each step. Samples were polymerized at 70°C overnight. Resin-embedded samples were sectioned using a Leica EM UC7 ultramicrotome (Leica, Wetzlar, Germany) prior to visualization of cellular ultrastructure using a Hitachi 7100 Transmission Electron Microscope (Hatachi High-Tech, Tokyo, Japan) and identification of cellular structures that resembled lamellar bodies.

### Doxorubicin Assay

The function of ABCA-3 in primary nasal epithelial cells was measured using an adapted methodology to quantify the capacity of the cells to detoxify doxorubicin (22). Cells were treated with different concentrations (0.25–10 μM) of doxorubicin for 3 h in triplicate for 96-well plates or duplicate for 12-well plates as previously described. An untreated control was included for each experiment. After 3 h of treatment with doxorubicin, cells were washed with PBS to remove residual doxorubicin and returned to nasal epithelial cell growth medium without epidermal growth factor. Cells were then incubated for a further 24 h at 37°C and 5% CO_2_ before cell viability or cytotoxicity was assessed. Initially, cell viability was assessed using MTS colorimetric assay (CellTiter 96® AQueous one solution cell proliferation assay; Promega, Madison, WI, USA) as per the manufacturer's instructions. Specific concentrations of doxorubicin were then selected to repeat cell exposure experiments to measure LDH release (CytoTox 96® colorimetric assay; Promega, Madison, WI, USA) and quantify the proportion of live cells using Calcein blue viability staining by flow cytometry (BD LSRFortessa™) according to manufacturer's instructions. Results were normalized to the untreated control.

### Statistical Analysis

Data were first tested for normality using the Kolmogorov–Smirnov and Shapiro–Wilk tests to determine whether parametric or non-parametric statistical analysis was appropriate. Where non-parametric tests were required or the sample size was small, Mann–Whitney tests were used to compare two groups of *n* > 3 replicates. Unpaired, two-tailed Student's *t*-tests were used to compare two groups of *n* = 3 replicates or when data passed the standards for normality. Where multiple groups were compared, the Kruskal–Wallis test was used with the Dunn *post-hoc* test to correct for multiple comparisons. Two-tailed tests were used for all statistical analysis. Data were presented as mean with error bars representing standard deviation depending on sample size. All *p* < 0.05 (^*^p < 0.05, ^**^p < 0.01, ^***^p < 0.001) were considered significant. GraphPad Prism version 7.04 was used for all statistical analysis.

## Results

### Gene Expression

**Figure 1:**
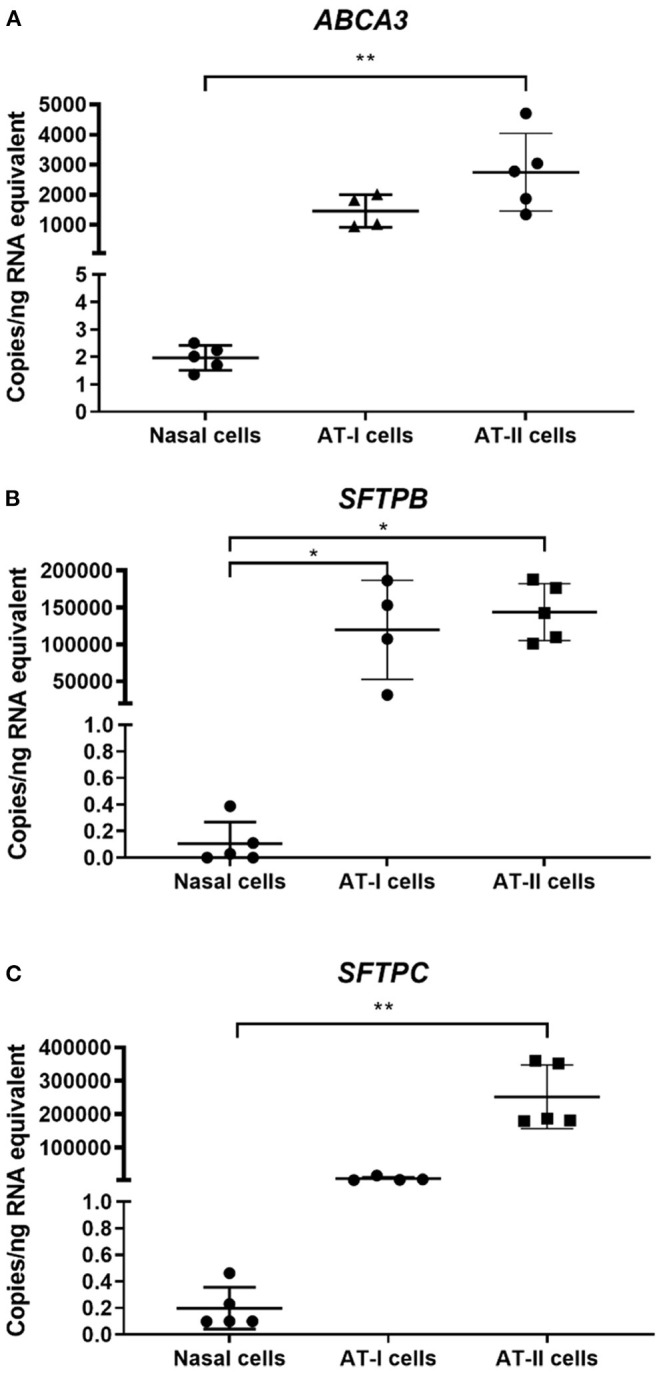
Gene expression in primary nasal cells. Concentration of target mRNA for *ABCA3* **(A)**, *SFTPB* **(B)**, *SFTPC* **(C)** in primary nasal cells (*n* = 5), AT-I cells (*n* = 4), and AT-II cells (*n* = 5) was determined by ddPCR and expressed as copies/ng of RNA equivalent. The expression of *ABCA3* was significantly lower in primary nasal cells compared to AT-II cells (*p* = 0.003). The expression of *SFTPB* was significantly lower in primary nasal cells compared to AT-I cells (*p* = 0.041) and AT-II cells (*p* = 0.010) while the expression of *SFTPC* in primary nasal cells was significantly lower compared to AT-II cells (*p* = 0.001). AT-I cells, AT-I cells; AT-II cells, AT-II cells; ^*^p < 0.05; ^**^p < 0.01.

Expression levels of target genes in primary nasal epithelial cells (*n* = 5) were compared to AT-I cells (*n* = 4) and AT-II cells (*n* = 5) (Figure 1). Expression levels of target genes are detailed in Table 1. The expression of *ABCA3* in primary nasal epithelial cells was significantly different to the other cell types investigated (*p* = 0.0003). Primary nasal epithelial cell expression of *ABCA3* was not significantly different compared to AT-I cells (*p* = 0.123) but significantly lower than AT-II cells (*p* = 0.003). Expression of *SFTPB* in primary nasal epithelial cells was significantly different to other cell types investigated (*p* = 0.0023). Primary nasal epithelial cell expression of *SFTPB* was significantly lower than AT-I cells (*p* = 0.041) as well as AT-II cells (*p* = 0.010). Expression of *SFTPC* in primary nasal epithelial cells was significantly different to the other cell types investigated (*p* < 0.0001). Expression of *SFTPC* in primary nasal epithelial cells was not significantly different to AT-I cells (*p* = 0.218) but significantly lower than AT-II cells (*p* = 0.001).

**Table 1:** Expression levels of target genes in copies/ng RNA equivalent.

**Cell type**	**Primary nasal cells**	**AT-I cells**	**AT-II cells**
ABCA3	1.97 ± 0.45	1.45 × 10^3^ ± 5.42 × 10^2^	2.74 × 10^3^ ± 1.29 × 10^3^
SFTPB	0.11 ± 0.16	1.19 × 10^5^ ± 6.71 × 10^4^	14.4 × 10^5^ ± 3.86 × 10^4^
SFTPC	0.20 ± 0.16	6.56 × 10^3^ ± 6.14 × 10^3^	2.52 × 10^5^ ± 9.52 × 10^4^

### Protein Expression

**Figure 2:**
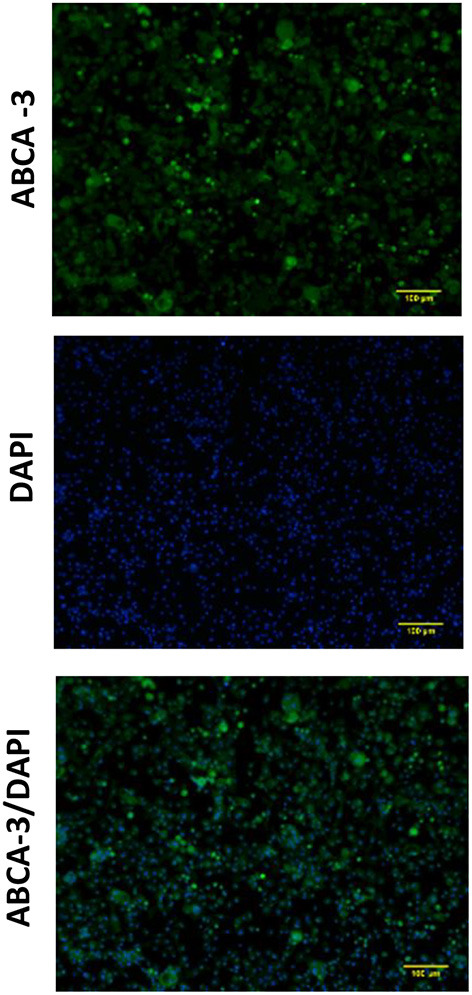
Expression of ABCA-3 in primary nasal epithelial cells. Expression of ABCA-3 was assessed in formaldehyde-fixed primary nasal epithelial cells cultured in chamber slides and visualized by fluorescent microscopy at 100 × total magnification. ABCA-3 was detected in primary nasal cells with a diffuse pattern of expression in the cytoplasm. DAPI was used as a nuclear stain. Scale bar 100 μm. Representative images are shown.

**Figure 3:**
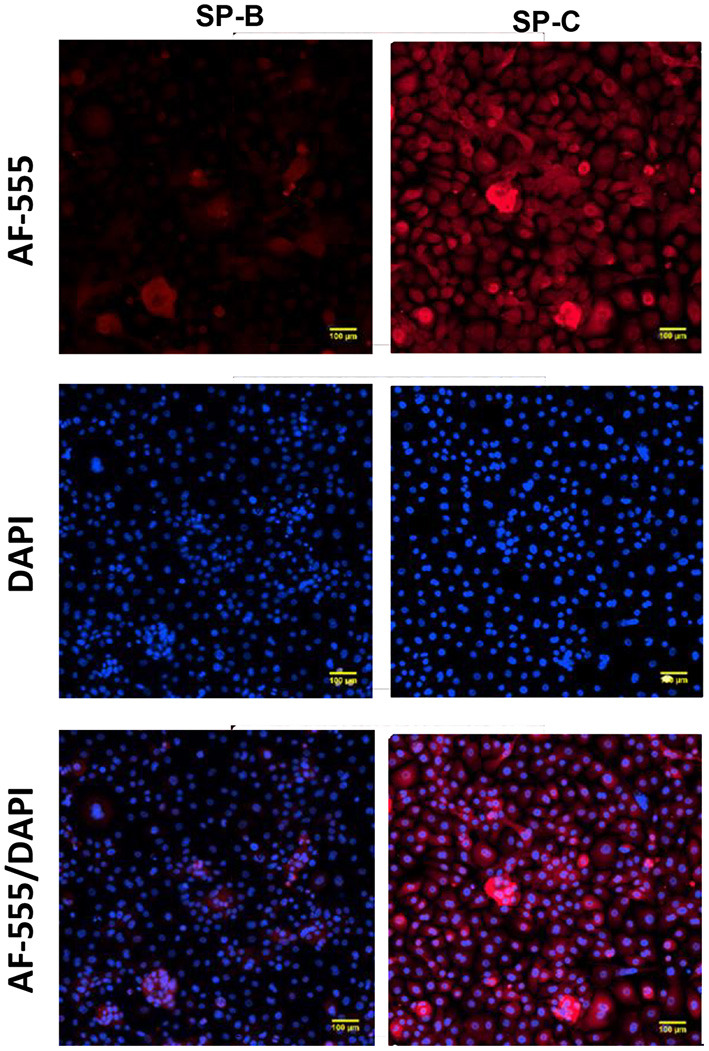
Expression of surfactant proteins in primary nasal epithelial cells. Expression of SP-B and SP-C was determined in formaldehyde-fixed primary nasal cells cultured in chamber slides and visualized by fluorescent microscopy at 100 × total magnification. A secondary antibody conjugated to AF-555 was used to detect primary antibodies bound to SP-B and SP-C. All protein targets were detected in the cytoplasm of primary nasal epithelial cells with a diffuse cytoplasmic pattern of expression and some cells staining more intensely than others. DAPI was used as a nuclear stain. Scale bar 100 μm. Representative images are shown.

**Figure 4:**
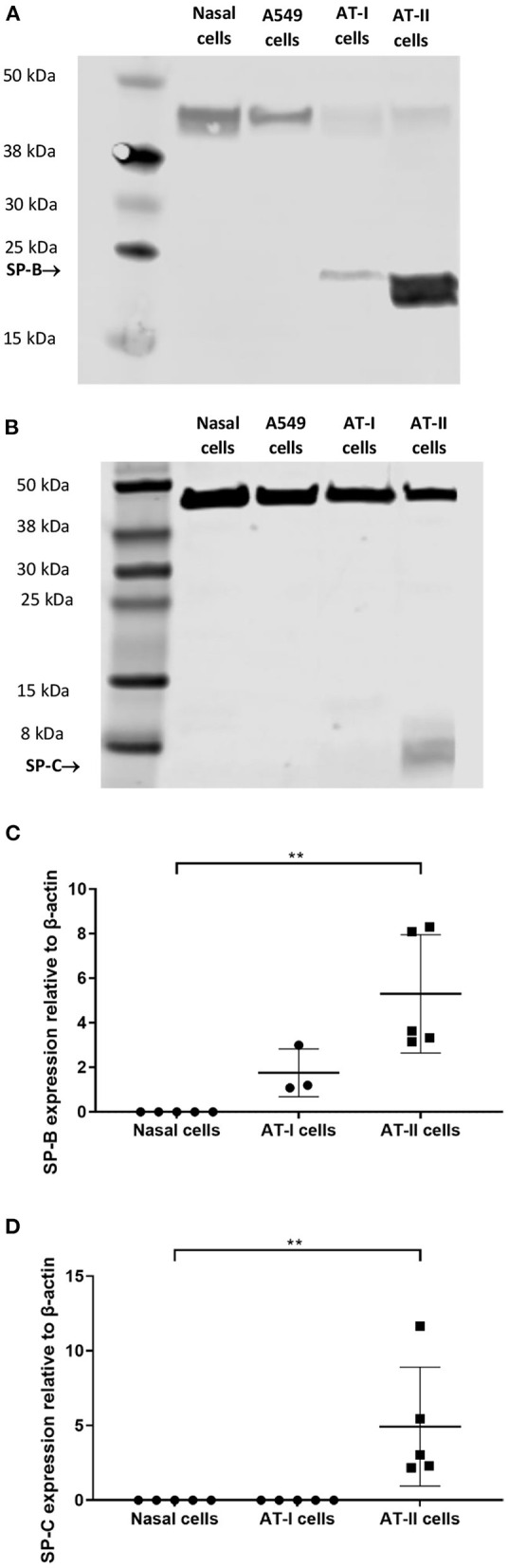
Western blot analysis on primary nasal cell lysates. Expression of SP-B and SP-C in primary nasal cell (*n* = 5), AT-I cells (*n* = 4), and AT-II cell (*n* = 5) samples was determined by western blot. An ~18 kDa protein was identified in type I and AT-II cells, which corresponded to the molecular weight of SP-B **(A)**. An ~7 kDa protein was identified in AT-II cells only that corresponded to the molecular weight of SP-C **(B)**. No SP-B or SP-C expression was detectable in primary nasal cell or A549 samples. The expression of SP-B **(C)** and SP-C **(D)** was quantified using integrated density measurements relative to the endogenous control, β-actin, which could be identified as a ~40 kDa band in all samples. Protein extracted from A549 cells was used as a comparator. Representative images are shown. AT-I cells, alveolar type I cells; AT-II cells, alveolar type II cells. ^**^p < 0.01.

Expression of ABCA-3 was evident in primary nasal epithelial cells (Figure 2), while SP-B and SP-C expression was detectable at low levels (Figure 3). All proteins exhibited a diffuse pattern of staining in the cell cytoplasm. However, there was heterogeneity in the intensity of the staining, with some cells fluorescing more strongly than others. There was also variability in the intensity of staining across cell cultures from different participants. Expression of SP-B or SP-C was not detectable in primary nasal epithelial cell samples using western blot (Figure 4). Expression of SP-B was detectable as an ~18 kDa protein band in AT-I cells [1.76 ± 1.07 arbitrary units (AU); *n* = 3] and AT-II cells (5.30 ± 2.65 AU; *n* = 5). After the removal of outliers, SP-B expression varied significantly across the different cell types (*p* < 0.0001). Expression of SP-C was detectable as a ~7 kDa protein band in AT-II cells (4.92 ± 3.98 AU; *n* = 5) only and varied significantly in different cell types (*p* = 0.001). The sizes of the SP-C and SP-B proteins detected by western blotting were equivalent to sizes previously described (23). The pattern of β-actin staining differed between non-reduced and reduced samples. The expression of ABCA-3 could not be quantified using Western blotting techniques with antibodies used for immunofluorescence staining.

### Organelle Ultrastructure

**Figure 5:**
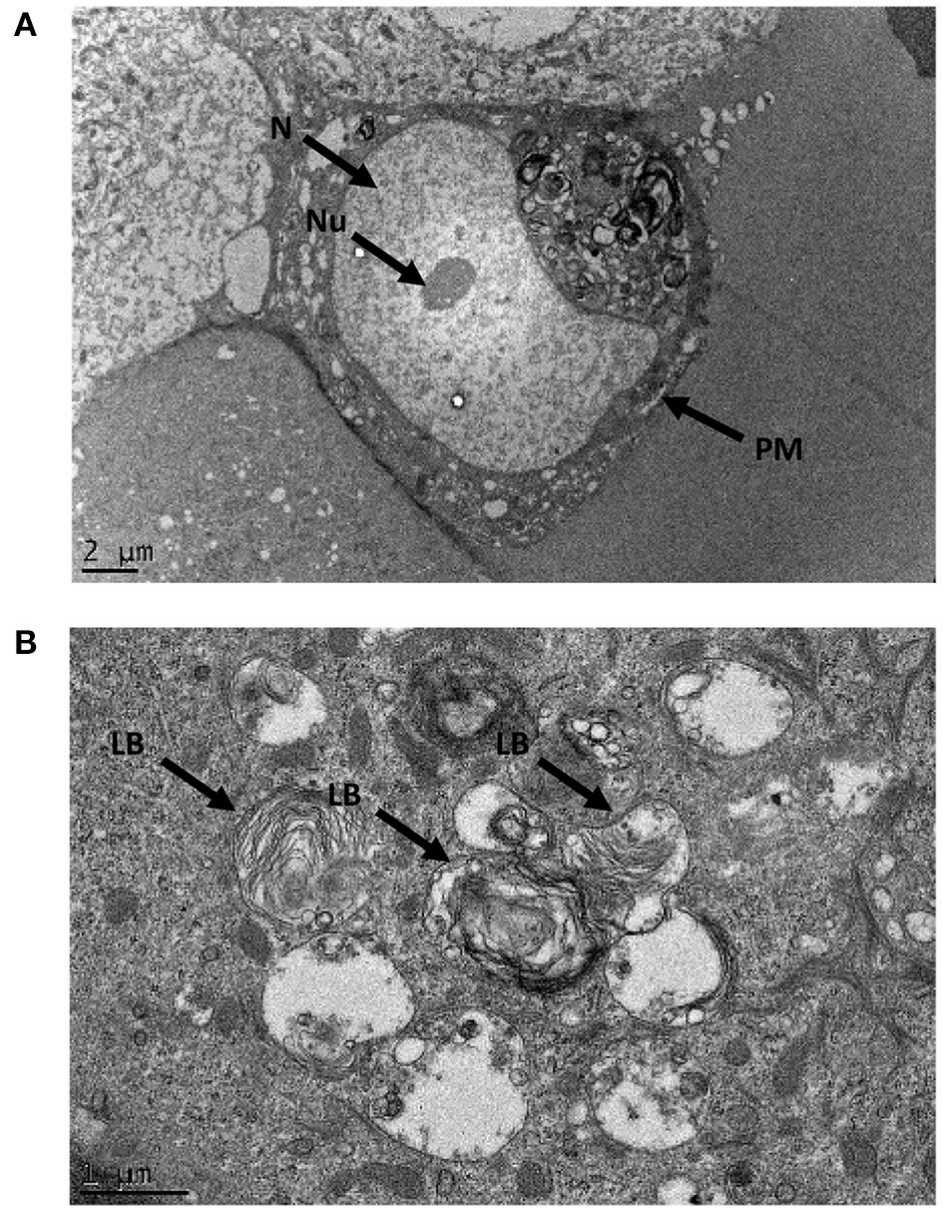
Ultrastructural morphology of nasal epithelial cells. Resin-embedded primary nasal epithelial cells were sectioned to a ~100 nm thickness and visualized using transmission electron microscopy at high magnification. At an ultrastructural level, lamellar body-like structures with osmiophilic, concentric membranes could be observed in electron micrographs at a cellular **(A)** and subcellular **(B)** level. N, nucleus; Nu, nucleolus; PM, plasma membrane; LB, lamellar body-like structure.

Electron micrographs of resin-embedded primary nasal epithelial cells demonstrated the presence of lamellar-body like structures. Intracellular organelles that have osmiophilic, concentric membranes could be identified in micrographs. While the structures resemble lamellar bodies, they appear to be structurally imperfect with disorganized lamellae. Representative images that show intracellular organelles at a cellular (Figure 5A) and subcellular level (Figure 5B) are depicted.

### Sensitivity to Doxorubicin

**Figure 6:**
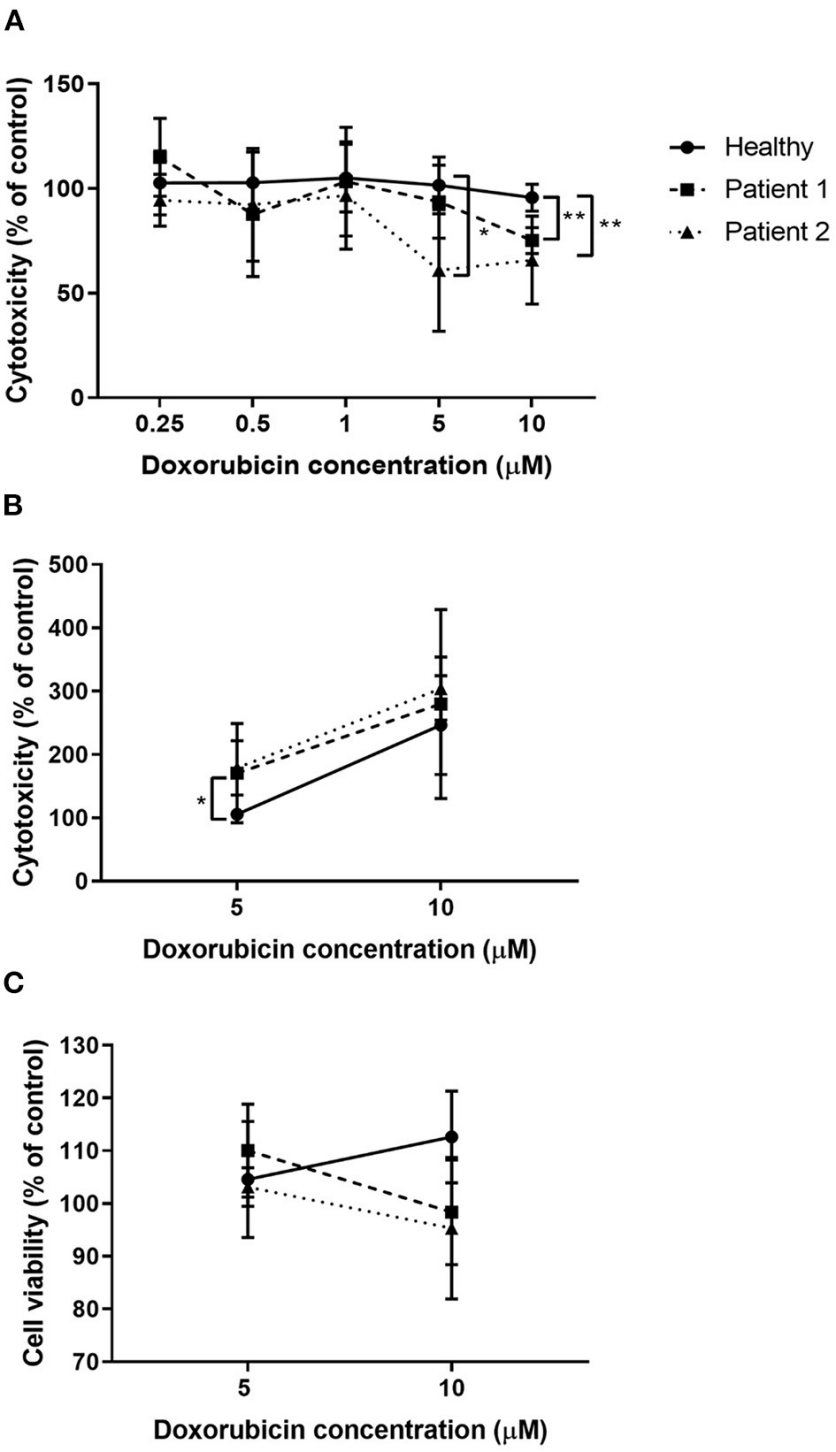
Doxorubicin treatment reduced viability of nasal epithelial cells from patients with ABCA-3 deficiency. Submerged cultures of primary nasal epithelial cells from participants with and without ABCA-3 deficiency were exposed to 0, 0.25, 0.5, 1, 5, or 10 μM of doxorubicin for 3 h at 37°C. Cell viability or cytotoxicity was measured 24 h post doxorubicin treatment. Viability, as measured using an MTS assay **(A)**, of nasal epithelial cells from Participant 2 was reduced by treatment with 5 μM doxorubicin (*n* = 3; *p* = 0.01) compared to healthy controls (*n* = 7). Doxorubicin at 10 μM significantly reduced viability of nasal epithelial cells from Participant 1 (*n* = 3; *p* = 0.002) and Participant 2 (*n* = 3; *p* = 0.006). To validate MTS assay results, submerged cultures of nasal epithelial cells from participants with and without ABCA-3 deficiency were exposed to 0, 5, or 10 μM doxorubicin for 3 h at 37°C. Doxorubicin at 5 μM but not 10 μM resulted in significantly increased cytotoxicity, measured using an LDH assay **(B)**, in nasal epithelial cells from Participant 2 (*n* = 3; *p* = 0.02) compared to healthy control nasal epithelial cells (*n* = 4) while doxorubicin treatment had no significant effect in nasal epithelial cells from Participant 1 (*n* = 3). No detectable differences in cell viability were observed between cell cultures from participants with and without ABCA-3 deficiency using calcein blue staining **(C)**. Sample size reflects biological replicates (healthy control cell cultures) or separate repeated experiments (participant cell cultures). Experiments were performed in triplicate. Data were normalized to the untreated control and expressed as a percentage. ^*^p < 0.05; ^**^p < 0.01.

Higher concentrations of doxorubicin reduced cell viability in primary nasal epithelial cells from individuals with ABCA-3 deficiency, compared to healthy controls in an assay-dependent manner (Figure 6). Primary nasal epithelial cells from Participant 1 demonstrated significantly reduced cell viability (*p* = 0.002) after treatment with 10 μM doxorubicin (75.13 ± 6.22%; *n* = 3) compared to healthy controls (95.63 ± 6.43%; *n* = 7) when measured using an MTS assay. Primary nasal epithelial cells from Participant 2 demonstrated significantly reduced cell viability after treatment with both 10 μM (65.78 ± 21.03%; *p* = 0.006; *n* = 3) and 5 μM doxorubicin (60.9 ± 29.1%; *p* = 0.01; *n* = 3) when compared to healthy controls (101.5 ± 13.59%; *n* = 7) measured using an MTS assay. Cell viability was not significantly reduced in primary nasal epithelial cells from participants with ABCA-3 deficiency by doxorubicin concentrations lower than 5 μM. To validate these findings, alternative measures of cell viability and cytotoxicity were utilized to quantify the response of primary nasal epithelial cells to treatment with 5 and 10 μM doxorubicin. Cytotoxicity measured by an LDH assay significantly increased in primary nasal epithelial cells from Participant 2 treated with 5 μM doxorubicin (178.9 ± 43.03%; *p* = 0.02; *n* = 3) compared to healthy controls (105.8 ± 7.92%; *n* = 4) but no significant difference was demonstrated when primary nasal epithelial cells were treated with 10 μM doxorubicin (304.2 ± 49.59%; *p* = 0.32; *n* = 3) with respect to healthy controls (246.5 ± 78.08%; *n* = 4). While 5 μM doxorubicin increased cytotoxicity, in primary nasal epithelial cells from Participant 1 (170.7 ± 78.48%; *n* = 3) compared to healthy controls, this result was not significant (*p* = 0.15). Similarly, 10 μM doxorubicin treatment did not significantly increase cytotoxicity in primary nasal epithelial cells from Participant 1 (279.7 ± 149.2%; *n* = 3) compared to the healthy control cells (*p* = 0.72). Calcein blue staining did not demonstrate a significant change in cell viability of primary nasal epithelial cells from Participant 1 (110 ± 8.80%; *p* = 0.51; *n* = 3) or Participant 2 (103.1 ± 3.63%; *p* = 0.84; *n* = 3) compared to healthy controls (104.6 ± 10.99%; *n* = 4) after treatment with 5 μM doxorubicin. Similarly, there was no significant difference in cell viability of primary nasal epithelial cells from Participant 1 (98.36 ± 9.94%; *p* = 0.10; *n* = 3) or Participant 2 (95.33 ± 13.41%; *p* = 0.10; *n* = 3) relative to healthy controls (112.6 ± 8.7%; *n* = 4) after treatment with 10 μM doxorubicin.

## Discussion

We demonstrated that *ABCA3* was expressed at a transcriptional and protein level in primary nasal epithelial cells, along with other surfactant-related genes *SFTPB* and *SFTPC*. However, expression of target genes was much lower in primary nasal epithelial cells compared to primary AT-II cells. Nevertheless, lamellar bodies, typically found in AT-II cells, were identified in primary nasal epithelial cells by TEM although the lamellar bodies were structurally imperfect. Furthermore, primary nasal epithelial cells cultured from participants with ABCA-3 deficiency had increased sensitivity to higher concentrations of doxorubicin when measured using an MTS assay. Taken together, these findings suggest the presence of detectable and functional ABCA-3 in primary nasal epithelial cells, which infers that the cell culture model could potentially be used as a tool to study ABCA-3 deficiency *in vitro* as a surrogate cell culture model for AT-II cells.

While the current study is the first report of ABCA-3 expression in cultured primary nasal epithelial cells, ABCA-3 has previously been detected in epithelial cells of the nasopharynx (Human Protein Atlas project). The results from the current study demonstrated that gene expression of ABCA-3 is low compared to that in AT-II cells. Additionally, a diffuse pattern of ABCA-3 staining seen by immunocytochemistry was observed as opposed to a vesicular pattern observed in AT-II cells (24). The difference in staining pattern between cell types may suggest that the function of ABCA-3 in primary nasal epithelial cells may be distinct from its function in AT-II cells. The wide distribution of expression of ABCA-3, at both a gene and protein level, over numerous tissue types (https://www.proteinatlas.org/ENSG00000167972-ABCA3) suggests that ABCA-3 may have roles beyond pulmonary surfactant homeostasis. Indeed, ABCA-3 has been implicated in lysosomal drug sequestration (25) and exosome shedding (26) in cancer, demonstrating ABCA-3's activity as an intracellular transporter protein for other endosomal compartments in the cell apart from lamellar bodies. In the current study, primary nasal epithelial cells with mutant ABCA3 had increased sensitivity to doxorubicin when assessed using an MTS assay. Similarly, HEK293 cells transformed with mutant ABCA3 were also more sensitive to doxorubicin (22). However, findings in the current study were assay-specific with alternative methods to measure cell viability and cytotoxicity in response to doxorubicin unable to validate results obtained by the MTS assay.

The different methods used in this study to assess viability and cytotoxicity in primary nasal epithelial cells in response to doxorubicin are based on distinct cell functions that may explain conflicting results between assays. The MTS assay relies on the production of a colored formazan product by the mitochondrial activity of living cells (27), while Calcein Blue staining relies on the intracellular esterase activity and membrane integrity of viable cells to produce a fluorescent product and contain it within the cell (28). The LDH assay quantifies the amount of extracellular LDH in the cell culture supernatant released by damaged cells and relies on the production of a colored formazan product indirectly through the enzymatic activity of LDH (29). Notably, doxorubicin is known to interfere with mitochondrial function, which may confound MTS assay results following doxorubicin treatment (30, 31). It is unknown if this effect would influence the validity of the assay in assessing ABCA-3 function in primary nasal epithelial cells. The use of Calcein Blue staining and flow cytometry to determine the proportion of viable cells of all cellular events may underestimate the cytotoxic effect of doxorubicin as debris from lysed cells and apoptotic bodies are not captured by this method.A more complex method of staining and gating by flow cytometry to determine cell populations undergoing different forms of cell death (32) may be required, although the choice of stain is critical due to the potentially confounding fluorescent properties of doxorubicin (33). Unlike Calcein Blue viability staining, the LDH assay has the capability to capture the full extent of necrosis as the extracellular LDH in the cell culture supernatant is directly related to the proportion of necrotic cells in the culture whether the cells are intact or not (34). However, the current study demonstrated marked variability in normalized cytotoxicity measurements between separate experiments for each participant for both doxorubicin concentrations tested. The lack of reproducibility of these results indicates that the use of the LDH assay to quantify doxorubicin-induced cytotoxicity may not be a reliable method to assess ABCA-3 function.

Due to the discrepancy in results between assays, it is essential to determine a reliable method to assess ABCA-3 function. To validate the findings using the doxorubicin detoxification assay, alternative methods could potentially be explored. The ATP hydrolysis activity of ABCA-3 has previously been used as a measure of ABCA-3 function (35) and may be worth testing although it is debatable whether the ATPase activity measured could be specifically attributed to ABCA-3. Other assays described in the literature as ABCA-3 functionality assays may be suitable for cell culture models that have lamellar body-like intracellular vesicles. Qualitative assessment of lamellar body morphology by electron microscopy could also be used to evaluate ABCA-3 function as previously performed (22) although this method cannot be applied to a high throughput context. Alternatively, quantitative assessment by confocal microscopy of lipid filling and volume of intracellular vesicles with the use of fluorescently-labeled TopFluor phosphatidylcholine could be adapted from studies in A549 cells transformed with exogenous ABCA3 (13). Whether these assays can be adapted to primary nasal epithelial cells to demonstrate functional rescue by potential therapeutic agents, such as small molecular correctors or potentiators (12, 36), in cells with mutant ABCA3 requires further investigation.

The current study is the first to report the presence of lamellar bodies in cultured primary nasal epithelial cells. As the lamellar bodies identified appear to be structurally imperfect, further validation would be necessary to assert that the subcellular structures seen here are functionally equivalent to the lamellar bodies found in AT-II cells with a role in surfactant biology. Immunogold labeling could be employed to demonstrate that lamellar body-associated proteins such as ABCA-3 (1), CD63, and lysosomal associated membrane protein 3 (LAMP-3) (37) are co-localized with the structures seen by TEM. Furthermore, staining with Lysotracker (38) and Nile red (39), coupled with high-resolution microscopy, could determine whether the vesicles are acidic and lipophilic, which provide further evidence that the structures are the functional equivalent to lamellar bodies in AT-II cells.

The lack of substantial expression of key AT-II cell markers, *SFTPB* and *SFTPC* at a transcriptional level compared to expression levels in AT-II cells, suggest that the role of primary nasal epithelial cell cultures as a model for alveolar disease may be limited. Mature SP-B and SP-C, as the hydrophobic surfactant proteins associated with pulmonary surfactant, are essential for normal lung function and ABCA-3 deficiency is sometimes associated with reduced or absent mature SP-B and SP-C (40, 41) or altered levels of the mature forms relative to their precursors (6, 40). The complete biosynthesis of mature SP-B and SP-C from their respective precursors is thought to be an exclusive function of AT-II cells (42–44). While there are limited reports that SP-B is present in the human nasal mucosa (16) there are no reports of SP-C expression in the nasal epithelium. No SP-B or SP-C expression was detected in primary nasal epithelial cells in the current study using western blotting. However, in contrast, mature SP-B and SP-C were detected in primary nasal epithelial cell cytoplasm by immunocytochemistry. The discrepancy between the western blotting and immunocytochemistry results for SP-B and SP-C expression requires further investigation.

In summary, while the primary nasal epithelial cells used in the current study differ significantly from the AT-II cell phenotype, they could still be potentially used as a surrogate cell culture model in the context of ABCA-3 deficiency to an extent. The results from the current study demonstrate that ABCA3 is expressed at a gene and protein level in primary nasal epithelial cells. Further validation is required to confirm that the lamellar bodies detected by TEM are functional equivalents of lamellar bodies in AT-II cells. For a primary nasal epithelial cell culture model to be ideal for the development of therapeutics, an appropriate functional assay would need to be established to quantify ABCA-3 function in response to drug treatment. The ability of the doxorubicin detoxification assay to discern between primary nasal epithelial cells from participants with and without ABCA-3 deficiency was largely dependent on the method used and the specificity of this result to the function of the ABCA-3 protein could not be assessed in the current study. Further research is required to determine a suitable ABCA-3 functionality assay that is specific, sensitive and robust enough to use in primary nasal epithelial cells as a screening tool for therapeutics for ABCA-3 deficiency. Additionally, other experimental models that more accurately recapitulate the AT-II cell phenotype, such as AT-II-like cells derived from induced pluripotent stem cells or lung-derived adult tissue stem cells, may complement results found in primary nasal epithelial cells.

## Data Availability Statement

The original contributions presented in the study are included in the article/Supplementary Material, further inquiries can be directed to the corresponding author/s.

## Ethics Statement

The studies involving human participants were reviewed and approved by Princess Margaret Hospital Human Research Ethics Committee and St John of God Ethics Committee. Written informed consent to participate in this study was provided by the participants' legal guardian/next of kin.

## Author Contributions

NS performed the experimental work and completed first draft of the manuscript. AS, AK, SS, SW, and SF were involved in project conceptualization and manuscript review. All authors contributed to the article and approved the submitted version.

## Funding

NS was supported by an Australian Government Research Training Program Scholarship and a Perth Children's Hospital Foundation Ph.D. Top-Up Scholarship. AK is a Rothwell Family Fellow and SS is an NHMRC Practitioner Fellow. AS is supported by an NHMRC Investigator Grant (APP1193796).

## Conflict of Interest

The authors declare that the research was conducted in the absence of any commercial or financial relationships that could be construed as a potential conflict of interest.

## Publisher's Note

All claims expressed in this article are solely those of the authors and do not necessarily represent those of their affiliated organizations, or those of the publisher, the editors and the reviewers. Any product that may be evaluated in this article, or claim that may be made by its manufacturer, is not guaranteed or endorsed by the publisher.
