# Supplementary material for: Primary Nasal Epithelial Cells as a Surrogate Cell Culture Model for Type-II Alveolar Cells to Study ABCA-3 Deficiency
Source: Front Med (Lausanne). 2022 Feb 21;9:827416. doi: 10.3389/fmed.2022.827416 (PMC8899037; doi:10.3389/fmed.2022.827416)
Supplement: Supplementary file 1 [file Table_1.DOCX]

**Supplementary Data Table 1. Raw values obtained by ddPCR for gene expression of lung markers across cell culture type.**

| **Cell culture type** | **Sample identifier** | **Gene expression (copies/ng RNA equivalent)** | | | | | | | |
| --- | --- | --- | --- | --- | --- | --- | --- | --- | --- |
|  |  | *ABCA3* | *SFTPB* | *SFTPC* | *NKX2.1* | *ETV5* | *SOX9* | *SOX2* | *TP63* |
| A549 cell line | A549_1 | 89.4 | 0.19 | 0.96 | 0.11 | 446.65 | 342.05 | 47.96 | 91.94 |
| A549 cell line | A549_2 | 182.63 | 0 | 0.16 | 0 | 211.89 | 171.97 | 74.35 | 140.08 |
| A549 cell line | A549_3 | 216.76 | 0 | 0.36 | 0.28 | 397.86 | 116.1 | 107.86 | 302.64 |
| A549 cell line | A549_4 | 317.44 | 0 | 0.14 | 0.34 | 667.42 | 185.3 | 256.2 | 236 |
| Primary Type I Alveolar cell | hAEPC 1 AT-1 | 1819.66 | 107297 | 2619.15 | 1549.64 | 241.26 | 23.26 | 34.78 | 352.1 |
| Primary Type I Alveolar cell | hAEPC 2 AT-1 | 1024.89 | 186644.2 | 15686.58 | 3389.18 | 422.37 | 9.22 | 22.43 | 53.41 |
| Primary Type I Alveolar cell | hAEPC 3 AT-1 | 950.83 | 31553.07 | 3337.37 | 278.28 | 395.31 | 78.69 | 17.21 | 356.22 |
| Primary Type I Alveolar cell | hAEPC 5 AT-1 | 2012.37 | 153253.8 | 4589.55 | 1585.85 | 308.13 | 89.37 | 20.36 | 665.24 |
| Primary Type II Alveolar cell | hAEPC 1 AT-2 | 1341.34 | 176498.2 | 359875.6 | 1780.79 | 706.69 | 170.58 | 134.25 | 17.95 |
| Primary Type II Alveolar cell | hAEPC 2 AT-2 | 4704.98 | 187861.5 | 352083.1 | 2554.3 | 608.7 | 143.68 | 78.82 | 179.24 |
| Primary Type II Alveolar cell | hAEPC 3 AT-2 | 3039.08 | 142816.4 | 180950.2 | 359.09 | 263.99 | 28.42 | 15.86 | 7.96 |
| Primary Type II Alveolar cell | hAEPC 4 AT-2 | 2774.17 | 110122.5 | 179337.8 | 439.52 | 518.8 | 55.9 | 49.56 | 89.99 |
| Primary Type II Alveolar cell | hAEPC 5 AT-2 | 1862.47 | 101181.2 | 186649.4 | 505.58 | 249.64 | 52.06 | 138.44 | 21.32 |
| Primary Nasal Epithelial cell | ERP0006N | 1.36 | 0.11 | 0.1 | 0 | 35.97 | 60.97 | 108.63 | 1251.35 |
| Primary Nasal Epithelial cell | ERP0009N | 1.71 | 0 | 0.1 | 0 | 79.04 | 63.04 | 63.85 | 1136.06 |
| Primary Nasal Epithelial cell | ERP0028N | 2.51 | 0 | 0.23 | 0 | 67.2 | 82.4 | 131.2 | 1476 |
| Primary Nasal Epithelial cell | ERP0029N | 2.25 | 0.03 | 0.1 | 0 | 44.2 | 62.38 | 97.13 | 1362.72 |
| Primary Nasal Epithelial cell | HAP230N | 2.02 | 0.39 | 0.46 | 0.06 | 80.88 | 81.35 | 131.42 | 1780.09 |
